# Apocynin Prevents Abnormal Megakaryopoiesis and Platelet Activation Induced by Chronic Stress

**DOI:** 10.1155/2017/9258937

**Published:** 2017-11-28

**Authors:** Leonardo Sandrini, Alessandro Ieraci, Patrizia Amadio, Maurizio Popoli, Elena Tremoli, Silvia S. Barbieri

**Affiliations:** ^1^Centro Cardiologico Monzino, IRCCS, Milan, Italy; ^2^Dipartimento di Scienze Farmacologiche e Biomolecolari, Università degli Studi di Milano, Milan, Italy

## Abstract

Environmental chronic stress (ECS) has been identified as a trigger of acute coronary syndromes (ACS). Changes in redox balance, enhanced reactive oxygen species (ROS) production, and platelet hyperreactivity were detected in both ECS and ACS. However, the mechanisms by which ECS predisposes to thrombosis are not fully understood. Here, we investigated the impact of ECS on platelet activation and megakaryopoiesis in mice and the effect of Apocynin in this experimental setting. ECS induced by 4 days of forced swimming stress (FSS) treatment predisposed to arterial thrombosis and increased oxidative stress (e.g., plasma malondialdehyde levels). Interestingly, Apocynin treatment prevented these alterations. In addition, FSS induced abnormal megakaryopoiesis increasing the number and the maturation state of bone marrow megakaryocytes (MKs) and affecting circulating platelets. In particular, a higher number of large and reticulated platelets with marked functional activation were detected after FSS. Apocynin decreased the total MK number and prevented their ability to generate ROS without affecting the percentage of CD42d^+^ cells, and it reduced the platelet hyperactivation in stressed mice. In conclusion, Apocynin restores the physiological megakaryopoiesis and platelet behavior, preventing the detrimental effect of chronic stress on thrombosis, suggesting its potential use in the occurrence of thrombosis associated with ECS.

## 1. Introduction

Thrombosis is a pathological event, which can affect both veins and arteries, whose clinical manifestation is associated with deep vein thrombosis, myocardial infarction, cerebral stroke, and pulmonary embolism. A number of studies have shown a strong link between cardiovascular disease and psychological stress. Thus, psychological stress has been recently included as risk factor for cardiovascular disease [1–3]. In particular, psychological/environmental stress, inducing hyperactivation of platelets and coagulation factors and modifying the normal fibrinolytic processes, triggers a hypercoagulable state [4], predisposing to cardiovascular disease.

Interestingly, modifications in the redox balance and in the reactive oxygen species (ROS) production and alterations of genes regulating antioxidant systems, including NADPH oxidase, occur in both chronic stress and cardiovascular disease [5–9].

Several studies showed that endothelial cells, leukocytes, vascular smooth muscle cells, fibroblasts, and platelets are important sources of ROS after vascular injury and that these ROS can regulate platelet activation [10, 11], triggering a vicious circle that leads to thrombosis. However, the mechanisms by which chronic environmental stress predisposes to thrombosis and thereby to cardiovascular disease are not fully understood.

Therapeutic approaches targeting ROS production to reduce atherothrombotic risk and to prevent neurodegenerative diseases are developing [12–14]. One of the most promising antioxidant drugs in experimental models is Apocynin (4-hydroxy-3-methoxyacetophenone) [15]. Apocynin is an active principle with anti-inflammatory properties [16] isolated from the root of the medicinal plant *Picrorhiza Kurroa* (Scrophulariaceae) [17]. Apocynin affects ROS generation inhibiting NADPH oxidase enzyme activation, and it turns out to be a scavenger of hydrogen peroxide [15, 18].

In this study, we aimed at evaluating the beneficial effect(s), if any, of Apocynin in preventing thrombosis induced by chronic psychological/behavioral stress and at exploring the underlying mechanism(s).

## 2. Methods

### 2.1. Ethics Statement and Experimental Design

**Figure 1:**
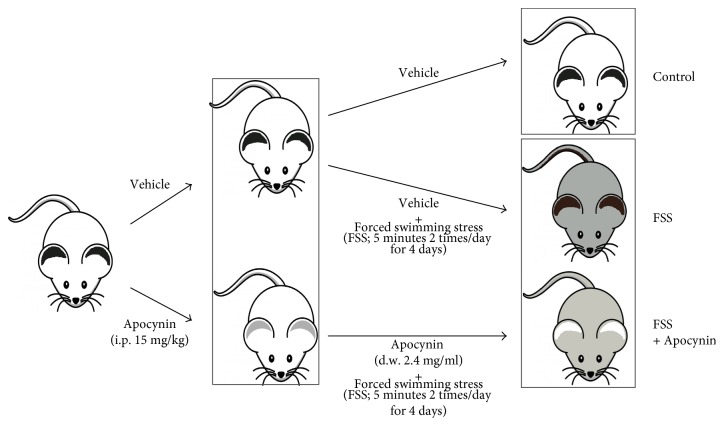
Design of the experiment: mice were randomized in 3 groups: control (vehicle), FSS (vehicle treatment and exposure to forced swim stress for 5 minutes two times/day as indicated), and FSS plus Apocynin (Apocynin treatment and exposure to forced swim stress for 5 minutes two times/day as indicated) and then sacrificed as indicated in the Methods 18 hours after the last section of stress.

The experimental groups consisted of FVB male mice (12–14 weeks old) supplied by Charles River and housed in a temperature-controlled, 12 h light/dark cycle environment with ad libitum access to water and fed on standard pellet diet. All experiments were approved by the National Ministry of Health-University of Milan Committee and of DGSA. Surgical procedures were performed in mice anesthetized with ketamine chlorhydrate (75 mg/kg; Intervet) and medetomidine (1 mg/kg; Virbac). Mice were randomized in different groups. Apocynin (Sigma-Aldrich; 15 mg/kg) or dimethylsulfoxide (DMSO) was intraperitoneally (i.p.) injected 1 h before start the first session of stress [8] and then added to the drinking water (300 mg/kg) for 5 days [19], corresponding to 2.4 mg/ml since our mice approximately drink 3–3.5 ml water/day. No side effects were reported during long time treatment (2–16 weeks) with Apocynin [19, 20]. The chronic forced swim stress (FSS) was performed as previously described [8]. Briefly, mice were individually placed in a 2000 ml glass beaker (height, 24 cm; diameter, 12 cm) filled with 1500 ml of room temperature water for 5 min twice a day for 4 consecutive days. Mice were then quickly dried with a towel and returned to their home cage. This model of stress is based on the well-established finding that repeated exposure to an inescapable stress, for which no active coping responses are available, induces depressive- and anxious-like behaviors in mice [21–23]. Interestingly, this model of stress is considered a chronic model because behavioral alterations are chronically maintained for more than 4 weeks [24–26]. Experiments were performed, and tissue, were collected 18 h after the last section of FSS (Figure 1).

Blood was collected into 3.8% sodium citrate (1 : 10 vol:vol) from anesthetized mice at time indicated. For plasma preparation, citrated blood was centrifuged at 3000 rpm for 20 min at 4°C to obtain plasma, which was immediately stored at −80°C until further analysis.

### 2.2. Whole Blood Counts

Blood was collected into sodium citrate from anesthetized mice by cardiac venipuncture, and the differential white blood cell and platelet count were performed on a Beckman Coulter AU480.

### 2.3. Rotational Thromboelastometry

Rotational Thromboelastography (ROTEM®) was used to measure coagulability in whole blood as previously described [27]. Briefly, citrated whole blood was added to a cuvette containing star-tem® solution or fib-tem® solution, and recording was started immediately and was allowed to proceed for 60 min according to the manufacturer's instructions. Clotting characteristics were analysed in terms of clotting time (CT, time necessary for the formation of a clot with a diameter of 2 mm) and clot formation time (CFT, kinetic of a stable clot formation by activated platelets and fibrin) and maximum clot firmness and maximum clot elasticity (MCF and MCE, measure of the consistence and elasticity of the clot, resp.) were collected.

### 2.4. Malondialdhyde and NADPH/NADP Analyses

Malondialdhyde (Cat. MBS269473) and NADPH/NADP (Cat. MBS169276) were measured in plasma and bone marrow, respectively, by ELISA kit (MyBioSource) according the to manufacturer's instructions.

### 2.5. Arterial Thrombosis Model

Experimental arterial thrombosis was induced as previously described [28]. The left carotid artery of anesthetized mice was dissected free and placed in the probe (model 0.7V, Transonic System) connected to transonic flow meter (Transonic T106). After blood flow stabilization (baseline flow constant for 7 min at least 0.8 ml/sec), a 1 × 1 mm strip of filter paper (Whatman N°1) soaked with FeCl_3_ (10% solution; Sigma-Aldrich) was applied over the carotid artery. After 3 min, the filter paper was removed, the carotid artery was washed with PBS, and the flow was recorded for 30 min. An occlusion was considered to be total and stable when the flow was reduced by >90% from baseline until the 30-minute observation time, with the flow during this period not changing by more than 1% from baseline per second.

### 2.6. Platelet–Leukocyte Aggregate Analysis

Platelet/monocyte and platelet/neutrophil aggregates were analysed as previously described [29]. Briefly, citrated blood was stimulated where indicated with ADP for 5 minutes and red blood cells were lysed by FACS Lysing solution (BD Biosciences); samples were stained with the anti-CD45, anti-CD41, and anti-CD14 or anti-Lys6G (eBiolegend, Cat. 103101, 133901, 150101, and 127601, resp.) and analysed by flow FACS “Novocyte 3000.” A minimum of 5000 events was collected in the CD14^+^ or Lys6G^+^ gate.

### 2.7. Platelet Studies

Washed platelets (WPs) were obtained from platelet-rich plasma (PRP), isolated following centrifugation at 100*g* for 10 min of citrated blood as previously described [30], with serial centrifugation and addition of 0.2 mM PGI_2_ and 0.01 mg/l apyrase. Platelet pellets were resuspended in HEPES-Tyrode's buffer (137 mM NaCl, 20 mM HEPES, 5.6 mM glucose, 0.35% bovine serum, 1 mM MgCl_2_, 2.7 mM KCl, and 3.3 mM NaH_2_PO_4_).

25 *μ*l of WPs (5 × 10^4^/*μ*l in HEPES-Tyrode's buffer supplemented with 1 mM CaCl_2_) was mixed with a saturating concentration of PE-conjugated JON/A (Emfret Analytic, Cat. M023-2) antibody, raised against the activated form of GPIIbIIIa (*α*II*βΙ*II integrin), or with anti-CD62P and FITC-conjugated antibody (P-selectin; BD Biosciences, Cat. 553744), and the mixture reacted with different concentrations of ADP or thrombin for 15 minutes at room temperature. The reaction was stopped by 400 μl ice-cold PBS, and samples were analysed within 30 minutes. Platelets were identified by forward and side scatter distribution and by anti-CD41 positivity.

Reticulated platelets (RP) were identified by the thiazole orange method [30]: 10 *μ*l of PRP was incubated with 390 *μ*l of thiazole orange (Retic-Count; BD Biosciences) or PBS as control and anti-CD41 at room temperature for 10 minutes, in the dark.

Immediately after incubation, samples were analysed by flow cytometry collecting 10000 CD41-positive events; the percentage of RP was recorded, and the absolute number of RP was calculated by multiplying by the platelet count.

### 2.8. Megakaryocyte Analyses

For single-cell suspensions from bone marrow, BM was flushed in PBS (Ca^2+^- and Mg^2+^-free) containing 3% BSA, 5.5 mM D-glucose, 10.2 mM trisodium citrate, and 10 *μ*M PGE_1_ were filtered through a sterile nylon mesh (70 *μ*m) and centrifuged (5 min, 200*g*). Cells were first resuspended in FACS Lysing solution to lyse red blood cells, then washed and resuspended in PBS plus 2% FBS, and stained for 20 minutes on ice with PE-conjugated CD41 (BD Biosciences, Cat. 558040) and FITC-conjugated CD61 (BD Biosciences, Cat. 561911) or FITC-conjugated CD42d (Emfret Analytics, Cat. M061-1). The percentage of CD41-positive (CD41^+^) and CD61-positive (CD61^+^) cells or of CD41-positive (CD41^+^) and CD42d-positive (CD42d^+^) cells was evaluated by FACS on a FITC gate, and PE was set at 1% on samples stained with the control antibody [30]. CD41^+^/CD61^+^ was used as marker of total megakaryocyte, and CD41^+^/CD42d^+^ as marker of mature megakaryocytes.

For ROS detection, single-cell suspensions from bone marrow were stained with PE-conjugated CD41 and incubated with 20 *μ*M H_2_DCF-DA (2′,7′-dichlorodihydrofluorescein diacetate, Sigma-Aldrich) for 15 minutes at 37°C and 5% CO_2_, then samples were analysed by FACS. Live events were collected on the basis of side scatter plots. MKs were gated by identification of CD41-PE-positive cells [31].

Flow cytometry was performed on a flow FACS “Novocyte 3000,” collecting 3000 events per sample.

### 2.9. Bone Marrow Histology

Immunohistochemistry was performed on BM as previously described [30]. Tissues were fixed overnight in 4% formalin, embedded in paraffin, cut at 3 *μ*m, and mounted on polarized slides. BM samples were decalcified in 10% EDTA, pH 8 for 10 days before paraffin embedding. Sections were stained in Hematoxylin and Eosin (H&E). Slides were dewaxed, rehydrated, washed in PBS containing 0.1% Triton X100 for 15 minutes, and treated with 3% H_2_O_2_ for 10 minutes to block endogenous peroxidase. Antigen retrieval was performed with 0.01 M citrate buffer (pH 6) in a water bath at 98°C for 10 or 30 minutes according to the antibody used. Slides were incubated overnight at 4°C with the following primary antibodies: anti-NOX1 polyclonal antibody (1 : 200, GeneTex, Cat. GTX103888) or with rabbit preimmune IgGs (Vector Laboratories, Cat. I-1000) as negative controls. For immunoperoxidase, biotinylated anti-rabbit secondary antibody (Dako, Glostrup, Denmark, Cat. E0432) and then streptavidin/HRP (Dako, Glostrup, Denmark, Cat. P0397) were used and developed with the DAB substrate kit (Sigma-Aldrich). Slides were lightly counterstained with hematoxylin and observed and digitalized by a Zeiss Axioskop (Carl Zeiss) equipped with an intensified charge-coupled device (CCD) camera system (Photometrics). The number and area of megakaryocytes were evaluated in hematoxylin and eosin stained sections by counting 5–7 40x microscopic fields for each tissue sample [32].

### 2.10. Statistical Analysis

Statistical analyses were performed with GraphPad Prism 6.0 and SAS versus 9.4 software (SASA Institute). Data were analyzed by one- or 2-way ANOVA with repeated measures for main effects of treatment and time or stimuli, followed by a Dunn's test or a Bonferroni post hoc analysis as appropriate. Values of *p* < 0.05 were considered statistically significant. Data are expressed as mean ± SEM. For each box-plot, the center line illustrates the median, and box limits indicate the 25th and 75th percentiles.

## 3. Results

### 3.1. Effect of Apocynin on Chronic Stress and Oxidative Stress

**Figure 2:**
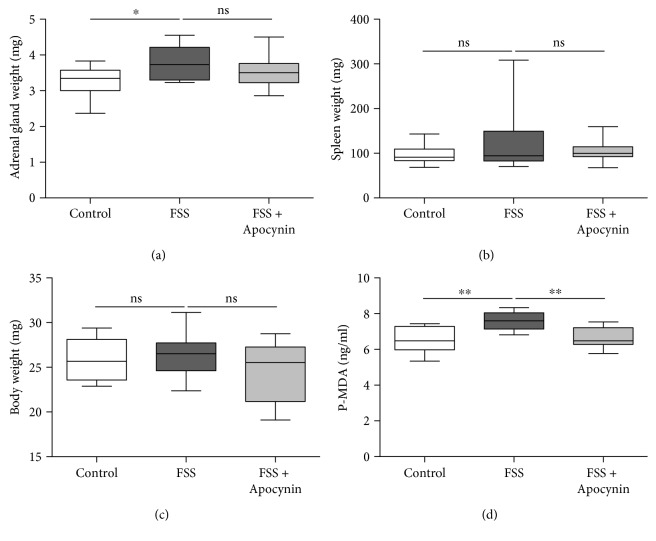
Forced swimming treatment for 4 days was sufficient to increase oxidative stress. Weight of (a) adrenal gland, (b) spleen, and (c) body and (d) plasma malondialdehyde (P-MDA) levels, from control mice (treated with vehicle) and from mice exposed to forced swimming stress with vehicle (FSS) or with Apocynin (FSS + Apocynin). *n* = 12 mice/group. ^∗^*p* < 0.05, ^∗∗^*p* < 0.01, and ns: nonsignificant. For each box-plot, the center line illustrates the median and box limits indicate the 25th and 75th percentiles. Data were analyzed by the Kruskal-Willis test followed by a Dunn's multiple comparison test.

To assess whether the chronic forced swimming treatment for 4 days was sufficient to induce a stress-response, we weighted adrenal glands as an index of terminal phase of hypothalamic-pituitary-adrenal neuroendocrine system activation. As expected, adrenal gland weight was greater in the stressed group of mice compared to the control group (*p* < 0.05; Figure 2(a)). Apocynin treatment did not modify the adrenal gland hypertrophy induced by forced swimming stress (FSS; Figure 2(a)), suggesting that Apocynin does not change the physiological response to the stress protocol. No significant difference in spleen or body weight between control and stressed mice with or without Apocynin treatment was found (Figures 2(b) and 2(c)).

Next, we measured the plasma malondialdehyde (P-MDA) levels, as marker of oxidative stress. The P-MDA levels were higher in the FSS-group compared to the control (*p* < 0.01; Figure 2(d)). Interestingly, Apocynin administration restored the normal P-MDA levels in stressed mice (Figure 2(d)).

### 3.2. Apocynin Prevented Arterial Thrombosis Induced by Forced Swimming Stress (FSS)

**Figure 3:**
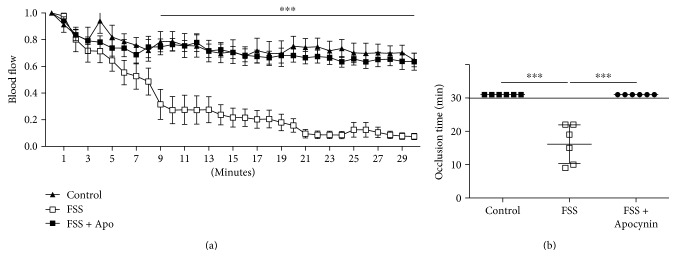
The propensity to arterial thrombosis induced by chronic stress is prevented by Apocynin treatment. (a) Blood flow in the carotid arteries of control mice (treated with vehicle) and from mice exposed to forced swimming stress with vehicle (FSS) or with Apocynin (FSS + Apocynin) expressed relative to the value before injury. Data shown are mean ± SEM and analyzed by 2-way ANOVA with repeated measures for main effects of treatment and time, followed by a Bonferroni post hoc analysis. (b) Time to thrombotic occlusion mice; horizontal bars indicate the mean value for each group. *n* = 6 mice/group. ^∗∗∗^*p* < 0.005. Data were analyzed by the Kruskal-Willis test followed by a Dunn's multiple comparison test.

FSS accelerated occlusion rates in FeCl_3_-injuried carotid arteries (Figure 3). In particular, FeCl_3_ application reduced dramatically the blood flow within 10 minutes and led to a stable occlusion in 100% of mice exposed to FSS, but it was unable to induce a stable occlusion in control mice within the same 30-minute observation period (Figure 3). In mice treated with Apocynin, blood flow was slightly but significantly reduced in the first 10 minutes after topical application of FeCl_3_ and remained constant over all the observation period (Figure 3). The total occlusion of carotid artery induced by FSS was completely prevented in all treated mice (Figure 3).

No significant difference in terms of basal levels of blood flow was noted between the animal groups (control: 0.616 ± 0.07 ml/min; FSS: 0.557 ± 0.10 ml/min and FSS + Apocynin: 0.543 ± 0.08 ml/min).

### 3.3. Effect of Forced Swimming Stress (FSS) on Blood Hemostasis

Thromboelastography analysis (ROTEM analyses) in whole blood provided evidence that FSS affected blood hemostasis by shortening the clotting formation time (CFT: control: 160.2 ± 10.39 sec and FSS: 90.67 ± 9.90 sec, *p* < 0.005) and increasing the maximum clot firmness (MCF: control: 60.33 ± 1.76 mm and FSS: 67.83 ± 1.6 mm, *p* < 0.05) and maximum clot elasticity (MCE: control: 154.5 ± 11.73 mm and FSS: 214.8 ± 16.27 mm, *p* < 0.05). In contrast, similar coagulation time in na-tem test (CT: control: 293.7 ± 15.38 sec and FSS: 235.3 ± 25.19 sec, *p* = 0.087) and comparable MCF in fib-tem test (MCF: control: 20.67 ± 1.91 mm and FSS: 23.50 ± 2.62 mm, *p* = 0.617) were detected in control and FSS groups.

These data suggest that chronic stress did not affect coagulation pathway and fibrinogen but instead, it promoted hyperactivation of platelets.

### 3.4. Effect of Apocynin on Circulating Cell Number and Platelet Function in Response to Forced Swimming Stress (FSS)

**Figure 4:**
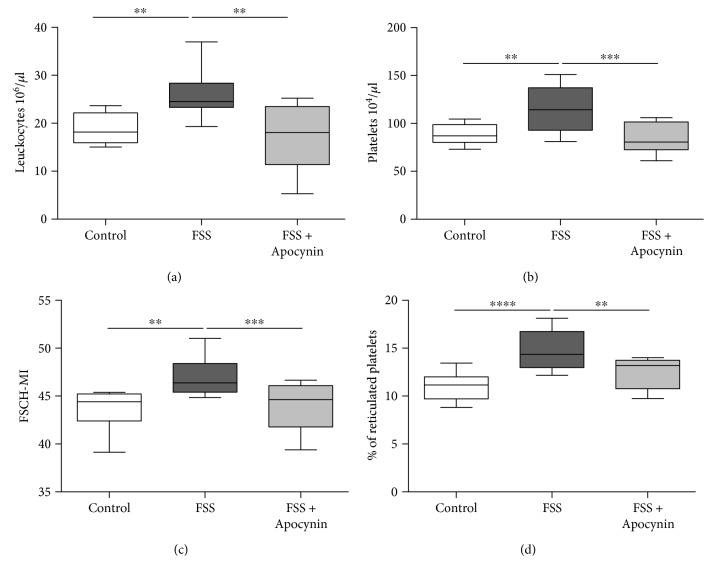
Modifications of circulating cells induced by chronic stressed are reduced by Apocynin treatment. Numbers of circulating (a) leukocytes and (b) platelets were counted, and (c) platelet size and (d) percentage of reticulated platelets were analysed by flow cytometry in control mice exposed to forced swimming stress (FSS) and treatment with or without Apocynin (FSS + Apocynin). *n* = 10 mice/group. ^∗∗^*p* < 0.01, ^∗∗∗^*p* < 0.005, and ^∗∗∗∗^*p* > 0.001. For each box-plot, the center line illustrates the median and box limits indicate the 25th and 75th percentiles. Data were analyzed by the Kruskal-Willis test followed by a Dunn's multiple comparison test.

As previously shown in mouse and human studies [33–35], FSS increased the number of circulating leukocytes (Figure 4(a), *p* < 0.01) and platelets (Figure 4(b), *p* < 0.01) and enhanced platelet size (Figure 4(c), *p* < 0.01) and the percentage of reticulated platelets (Figure 4(d), *p* < 0.001). Apocynin reverted these changes in stressed mice toward the restoration of the physiological number of leukocyte and platelet shape (Figure 4).

**Figure 5:**
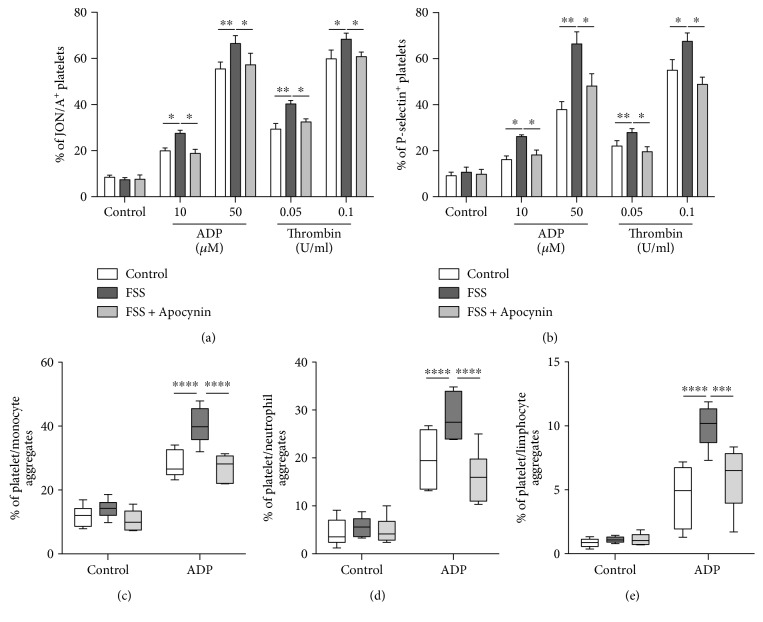
Apocynin decreases platelet activation induced by chronic stress. Flow cytometry analyses of (a) GPIIbIIIa activation (JON/A-PE antibody) and (b) P-selectin expression in washed platelets, and percentage of (c) platelet/monocyte, (d) platelet/neuthrophil, and (e) platelet/lymphocyte aggregates in whole blood of control mice (treated with vehicle) and of mice exposed to forced swimming stress with vehicle (FSS) or with Apocynin (FSS + Apocynin). *n* = 6 mice/group. ^∗^*p* < 0.05, ^∗∗^*p* < 0.01, ^∗∗∗^*p* < 0.005, and ^∗∗∗∗^*p* < 0.001. For each box-plot, the center line illustrates the median and box limits indicate the 25th and 75th percentiles. Data were analyzed by 2-way ANOVA with repeated measures for main effects of treatment and stimuli, followed by a Bonferroni post hoc analysis.

Interestingly, activation of integrin *α*IIb*β*3 (GPIIbIIIa) (Figure 5(a), *p* < 0.05) as well as the expression of P-selectin on platelet surface (Figure 5(b), *p* < 0.05) in response to different stimulus was higher in stressed mice compared to control. The greater expression of P-selectin went hand in hand with the enhanced percentage of platelet monocyte (Figure 5(c), *p* < 0.001), platelet/neutrophil (Figure 5(d), *p* < 0.001), or platelet/lymphocyte (Figure 5(e), *p* < 0.001) aggregates in response to ADP.

Remarkably, the platelet hyperreactivity induced by FSS was completely prevented by Apocynin treatment (Figure 5).

### 3.5. Effect of Apocynin on Megakaryopoiesis in Stressed Mice

To assess whether the thrombocytosis consequent to FSS exposure was associated to the abnormal megakaryopoiesis, bone marrow megakaryocytes were analyzed.

**Figure 6:**
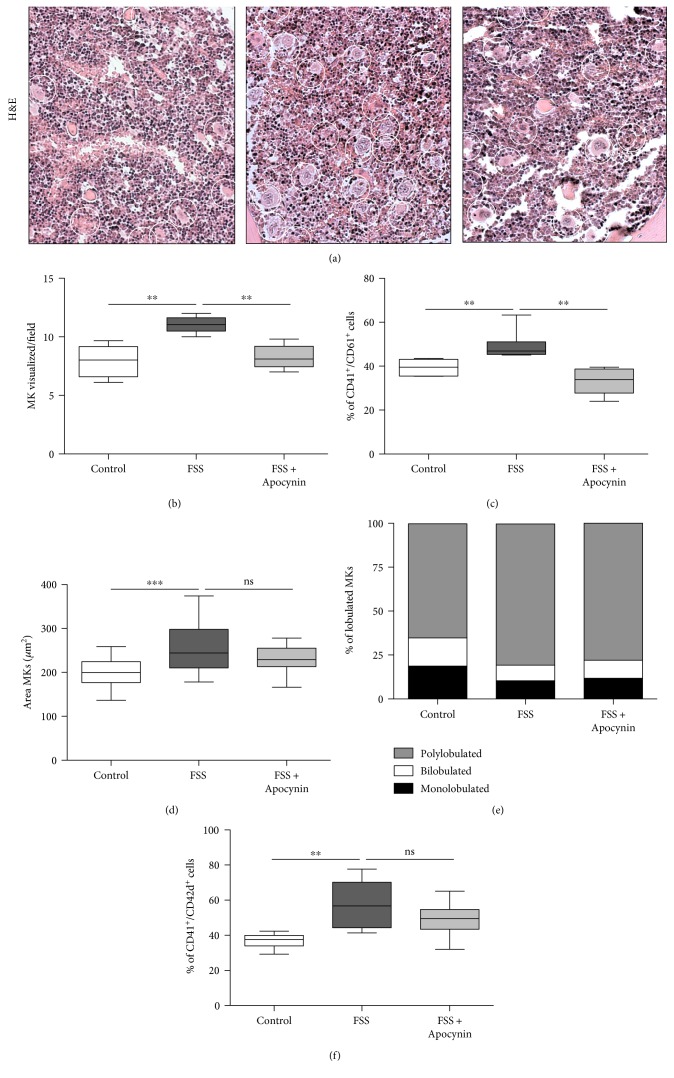
Apocynin partially reduces megakaryocyte alteration induced by chronic stress. (a) Hematoxylin and Eosine (H&E) staining of bone marrow from control mice (treated with vehicle) and from mice exposed to forced swimming stress with vehicle (FSS) or with Apocynin (FSS + Apocynin). Circle indicates MKs. (b) Quantification of panel (a) expressed as megakaryocytes per filed (40x magnification). Flow cytometric analyses: (c) percentage of CD41^+^/CD61^+^ cells and (f) percentage of CD41^+^/CD42d^+^ cells in bone marrow of mice. Analysis of (d) area and of (e) nuclear complexity in megakaryocytes from control, stress, and stress + Apocynin mice. *n* = 6 mice/group. ^∗∗^*p* < 0.01, ^∗∗∗^*p* < 0.005, and ns = nonsignificant. For each box-plot, the center line illustrates the median and box limits indicate the 25th and 75th percentiles. Data were analyzed by the Kruskal-Willis test followed by a Dunn's multiple comparison test.

Histopathological analysis of bone marrow section (Figure 6(a)) revealed a higher number of megakaryocytes (MKs) in stressed mice compared to control (FSS: 11.04 ± 0.28 MKs/field versus control: 7.93 ± 0.58 MKs/field, *p* < 0.01; Figure 6(b)), and flow cytometer analyses showed that FSS increased significantly the percentage of CD41^+^/CD61^+^ cells in bone marrow (Figure 6(c), *p* < 0.01). In addition, careful analyses of these sections revealed that megakaryocytes from stressed mice were larger (Figure 6(d), *p* < 0.005) and displayed greater nuclear complexity (Figure 6(e)) compared to control. In particular, higher percentage of polylobulated megakaryocytes (*p* < 0.001) and lower percentage of mononucleated (*p* < 0.01) or binucleated (*p* < 0.05) megakaryocytes were detected in bone marrow from stressed mice. The advanced maturation state of megakaryocytes from stressed mice suggested by morphological examination was sustained by the enhanced expression of lineage differentiation marker CD42d (Figure 6(f), *p* < 0.01). Interestingly, Apocynin decreased markedly the number of megakaryocytes (*p* < 0.01), but only slightly reduced the total area, without affecting their maturation state.

**Figure 7:**
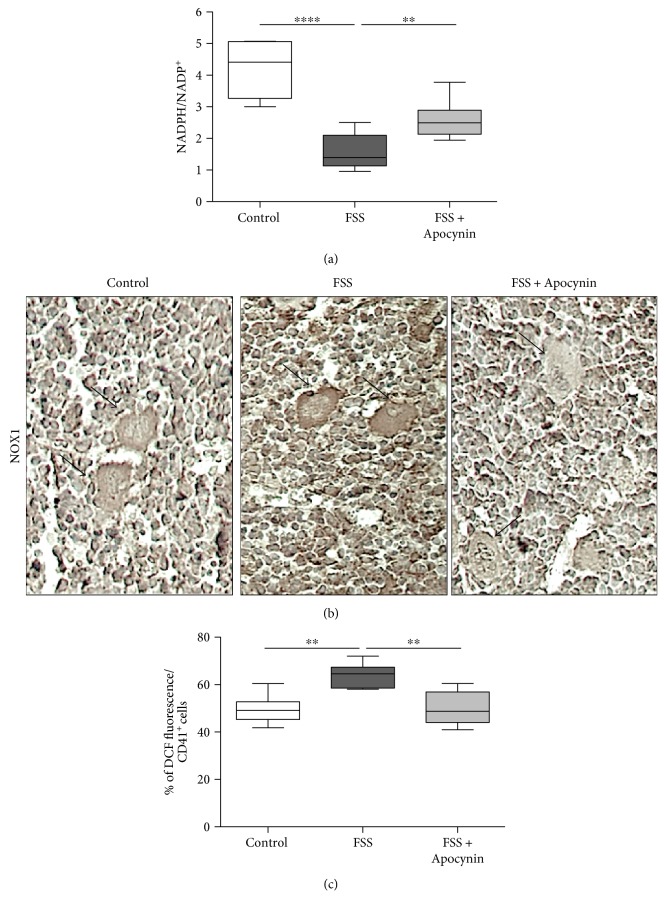
Apocynin prevents oxidative stress in megakaryocytes of stressed mice. Control mice and mice exposed to forced swimming stress (FSS) were treated with vehicle or with Apocynin (FSS + Apocynin), and then (a) NADPH/NADP^+^ ratio was measured in bone marrow. (b) Immunoperoxidase staining of NOX1 was performed in bone marrow. (c) Reactive oxygen species (ROS) levels were detected by H2DCF-DA in MKs, as described in Methods. *n* = 6 mice/group. ^∗∗^*p* < 0.01 and ^∗∗∗∗^*p* < 0.001. For each box-plot, the center line illustrates the median and box limits indicate the 25th and 75th percentiles. Data were analyzed by the Kruskal-Willis test followed by a Dunn's multiple comparison test.

Finally, we showed that FSS decreased the NADPH/NADP^+^ ratio (Figure 7(a)) and upregulated NOX1 expression in bone marrow, including megakaryocytes (Figure 7(b)). Interestingly, NOX1 is the most abundant NOX isoform in this type of cells [36]. In addition, FSS specifically induced reactive oxygen species (ROS) production in megakaryocytes (Figure 7(c), *p* < 0.01). As expected, Apocynin restored the physiological NADPH/NADP^+^ ratio and markedly decreased ROS generation and NOX1 expression induced by FSS (Figure 7).

## 4. Discussion

In this study, we show that Apocynin treatment, by preventing ROS generation, restores the physiological megakaryopoiesis and platelet function and reduces arterial thrombosis. Overall, these findings suggest that ROS play a key role in the susceptibility to thrombosis induced by chronic psychological/behavioral stress, affecting the production/activation of megakaryocytes, with consequent increase of both number and reactivity of circulating platelets.

Chronic environmental/psychological stress increases the hypothalamic-pituitary-adrenal axis activation [37] and induces a dysregulation in norepinephrine system that turns a homeostatic stress response into a pathological stress response [38]. Then, the observation that chronic environmental stress accelerates norepinephrine-mediated hematopoiesis promoting atherosclerotic plaque features associated with vulnerable lesions [35] well explained the correlation between psychological stress and acute myocardial infarction emerged from the INTERHEART study [39]. However, chronic environmental stress increases the risk for cardiovascular disease also promoting a prothrombotic state [40–42]. As a matter of fact, chronic stress increases the levels of tissue factor and of the plasminogen activation inhibitor-1 [40–42] and the number of circulating platelets [33]. Interestingly, here, we showed that 5 minutes of “forced swimming stress” session, performed twice a day for 4 consecutive days, is sufficient to induce a greater and more stable clot and to predispose mice to arterial thrombosis, without affecting fibrinogen and coagulation factor activation in agreement with data obtained by other stress models, such as restraint or foot shock stress [40, 43]. In addition, stressed mice not only have a higher number of circulating cells (leukocytes and platelets) but also have bigger, reticulated, and hyperresponsive platelets as previously reported in human studies [35, 44–46], which suggest modification in bone marrow milieu. Indeed, thrombopoiesis is a complex process resulting from many consecutive steps including proliferation and maturation of megakaryocytes [47]. Accordingly, we provide evidence that chronic stress increases the number of bone marrow megakaryocytes and it affects morphology as well. Megakaryocytes from stressed mice display an advanced maturation state, in terms of bigger size, greater nuclear complexity, and enhanced expression of CD42d antigen. In addition, megakaryocytes from stressed mice produce higher levels of ROS.

Previous studies have shown that both sympathetic transmitters (e.g., norepinephrine and epinephrine) and ROS promote proliferation of hematopoietic stem cells, their differentiation to megakaryocytes, and they induce megakaryocyte adhesion, migration, and proplatelet formation [33, 48]. Intriguingly, chronic stress increases norepinephrine levels in bone marrow [35], which in turn increases NADPH oxidase-derived superoxide in different type of cells [49, 50], suggesting a potential link between these cellular messengers under chronic stress condition. Moreover, we show that the NOX1 catalytic subunit, expressed in different type of cells, including macrophages, hematopoietic stem cells, and megakaryocytes [36, 51, 52], is upregulated by chronic stress in all bone marrow cells and not only in megakaryocytes.

Interestingly, previous experiments performed with different oxidase inhibitors suggest that the NADPH oxidase enzyme is the critical source of ROS in megakaryocytes [53] and that NADPH oxidase inhibition completely prevents the signaling cascade activation required for megakaryocyte differentiations [54], reducing their polyploidization [36]. However, the single deletion of NOX1 or of NOX2 alone, some of the subunits of NADPH oxidase, is not sufficient to modulate MK polyploidization [36] as provided by *in vivo* experiment, supporting the concept of a mutual compensation between the NOX isoforms.

Experimental and clinical data show that NADPH oxidase plays a key role also in platelet ROS generation and platelet activation [7, 55–57].

In line with these findings, we show here for the first time that Apocynin treatment prevents the detrimental effect of chronic psychological/behavioral stress, affecting production of ROS and leading to thrombosis, throughout the regulation of megakaryopoiesis and platelet activation.

In particular, the strong effect of Apocynin in the prevention of prothrombotic phenotype promoted by environmental stress may be the result of a pleiotropic effect of this compound on different cells and tissues (e.g., megakaryocytes, bone marrow milieu, platelets, and vessel walls). Indeed, the higher levels of NOX1 expression induced by chronic stress in bone marrow cells, including also megakaryocytes, are significantly reduced by Apocynin treatment. In addition, Apocynin prevents platelet activation and improves endothelial/vascular function [58, 59] reducing ROS by preventing their generation by the binding of NOXO1 and p47phox to p22phox and/or decreasing Rho kinases activity [60] and acting as radical scavenger [18]. However, Apocynin may affect platelet function also by a mechanism different from the abovementioned, which has not been well identified [61]. In particular, it is demonstrated that Apocynin might indirectly inhibit NADPH oxidase activity depleting its catalytic substrates such as NADPH [62] and decreasing the intracellular reduce/oxidase GSH/GSSG ratio [63].

## 5. Conclusion

Our data not only provide new important knowledge about the mechanisms by which chronic stress affects arterial thrombosis but also suggest that Apocynin is useful in the prevention of thrombosis associated to chronic environmental stress.
